# Supplementary figures and images for: SCGG: A deep structure-conditioned graph generative model
Source: PLoS One. 2022 Nov 21;17(11):e0277887. doi: 10.1371/journal.pone.0277887 (PMC9678307; doi:10.1371/journal.pone.0277887)

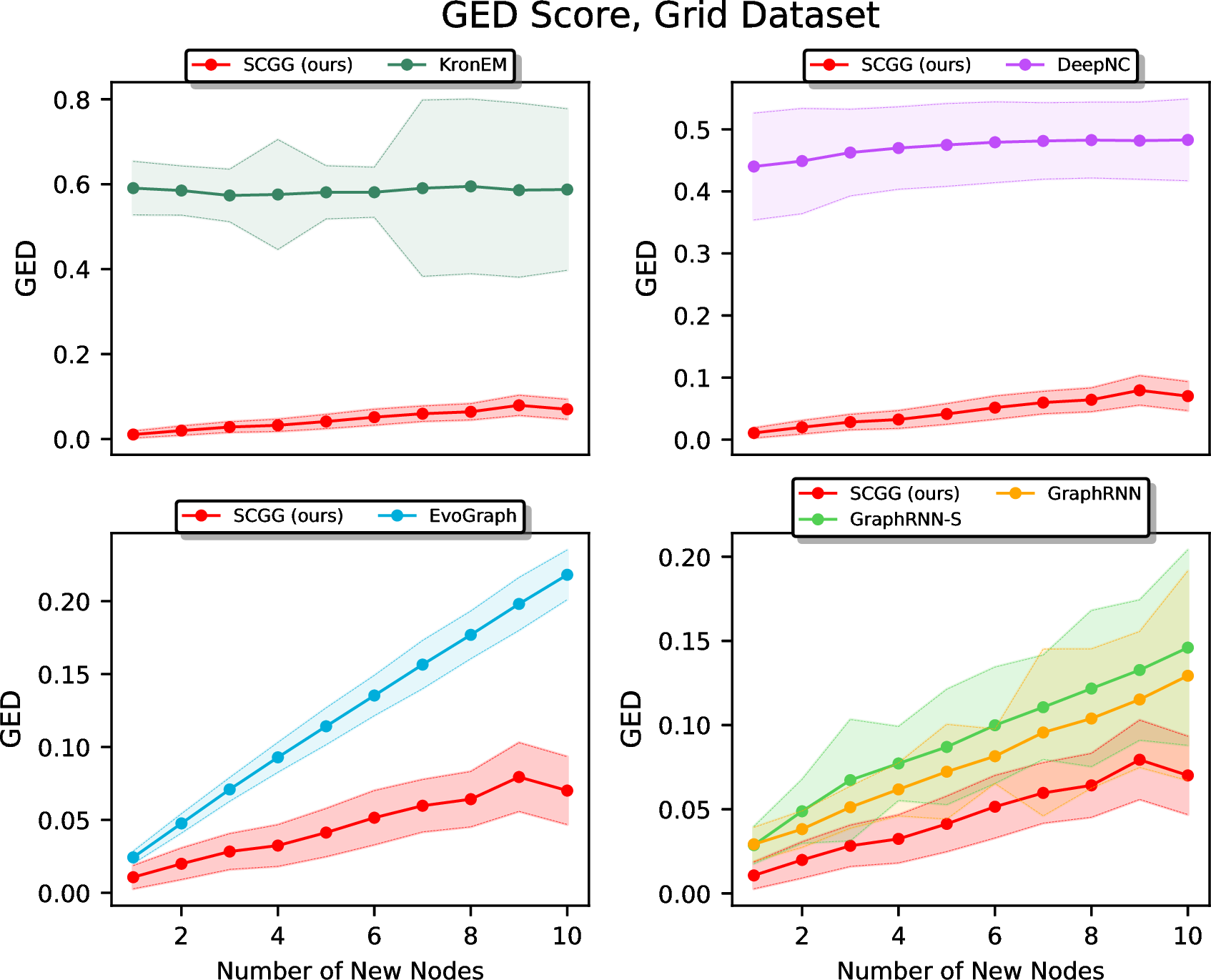

Supplement: S1 Fig — The results are reported in terms of GED (the lower the better) as a function of the number of new nodes (denoted by m) that are added to initial graphs (each represented by the notation G0 in the paper). (TIF) [file pone.0277887.s001.tif]

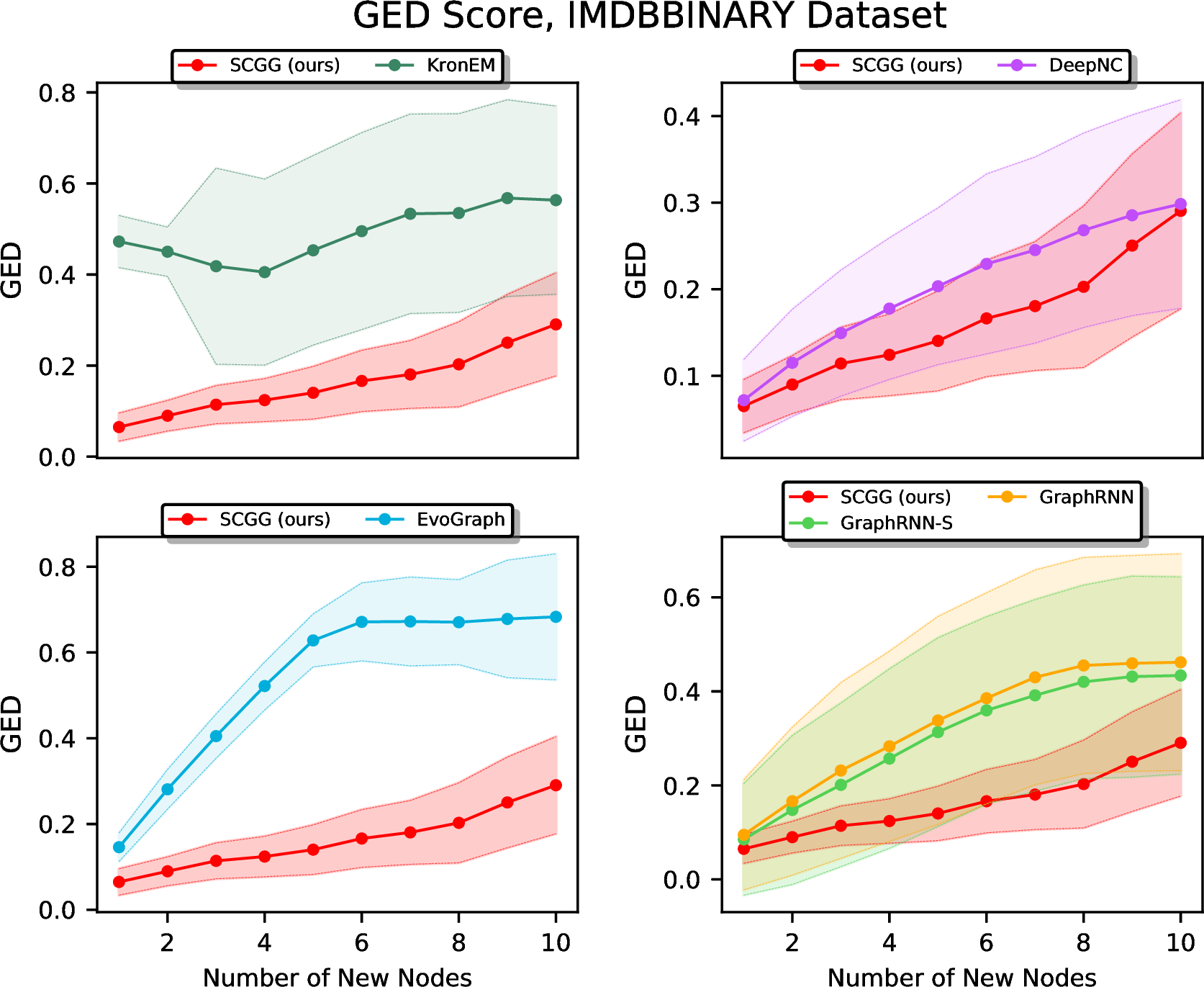

Supplement: S2 Fig — The results are reported in terms of GED (the lower the better) as a function of the number of new nodes (denoted by m) that are added to initial graphs (each represented by the notation G0 in the paper). (TIF) [file pone.0277887.s002.tif]

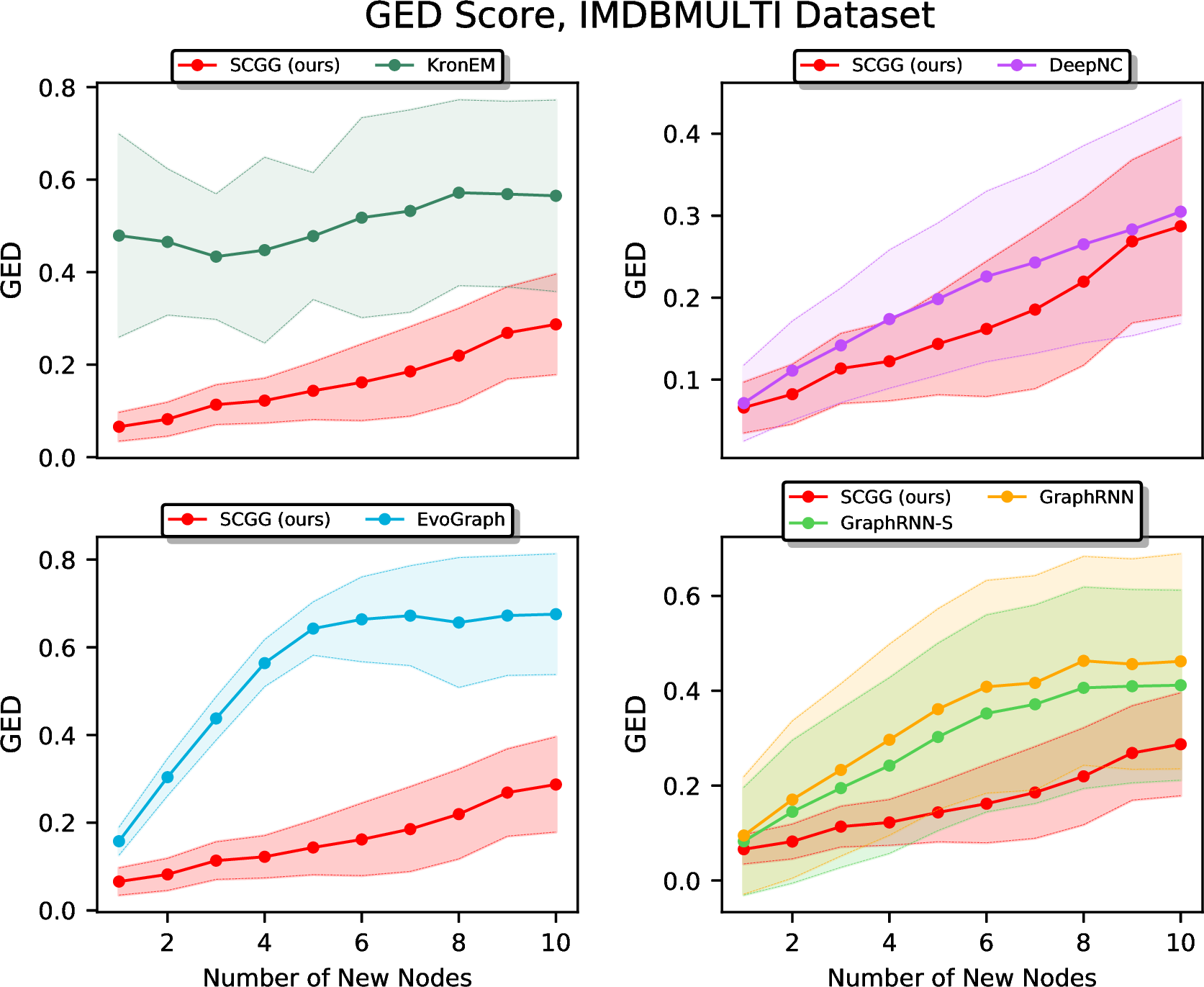

Supplement: S3 Fig — The results are reported in terms of GED (the lower the better) as a function of the number of new nodes (denoted by m) that are added to initial graphs (each represented by the notation G0 in the paper). (TIF) [file pone.0277887.s003.tif]

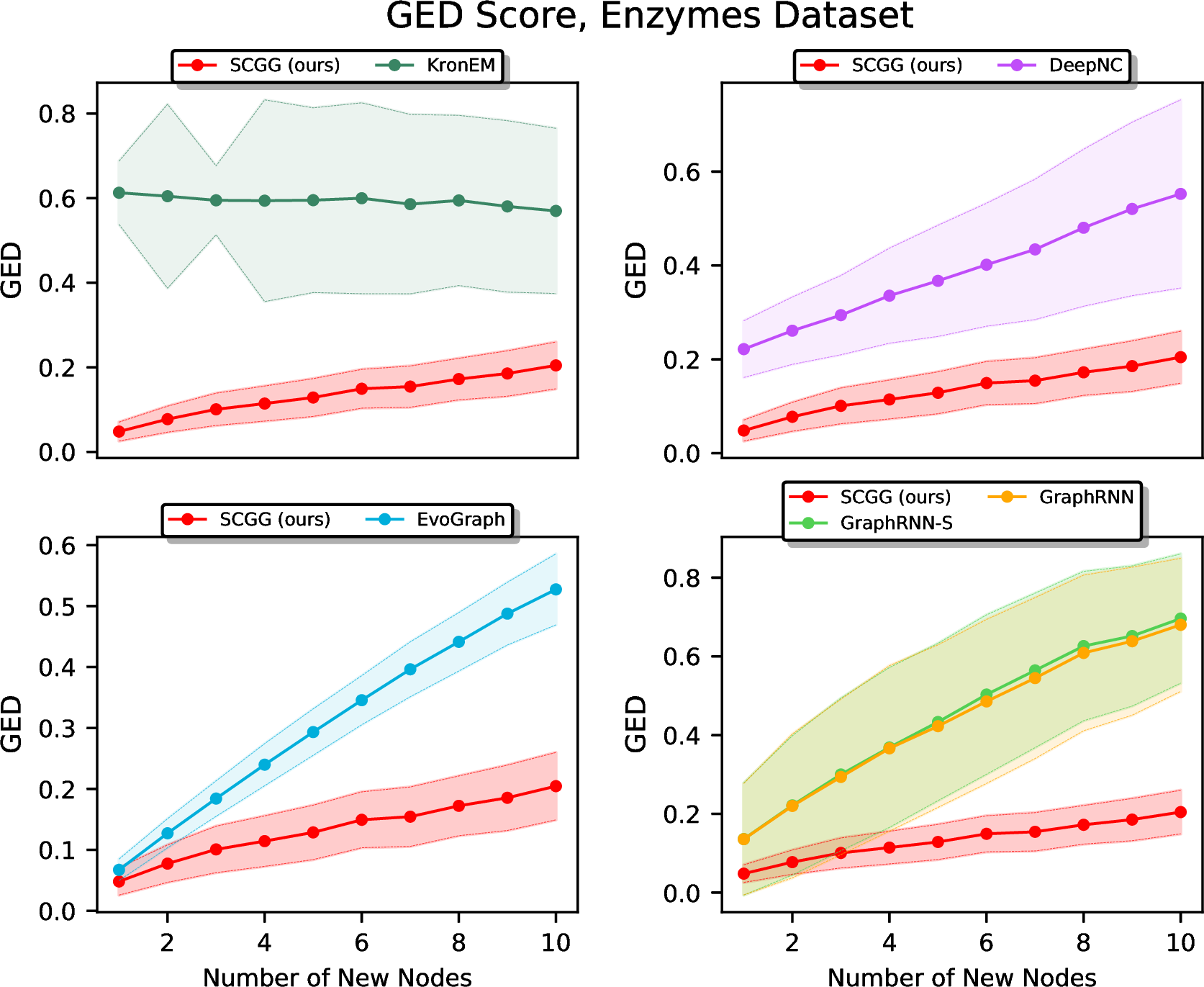

Supplement: S4 Fig — The results are reported in terms of GED (the lower the better) as a function of the number of new nodes (denoted by m) that are added to initial graphs (each represented by the notation G0 in the paper). (TIF) [file pone.0277887.s004.tif]

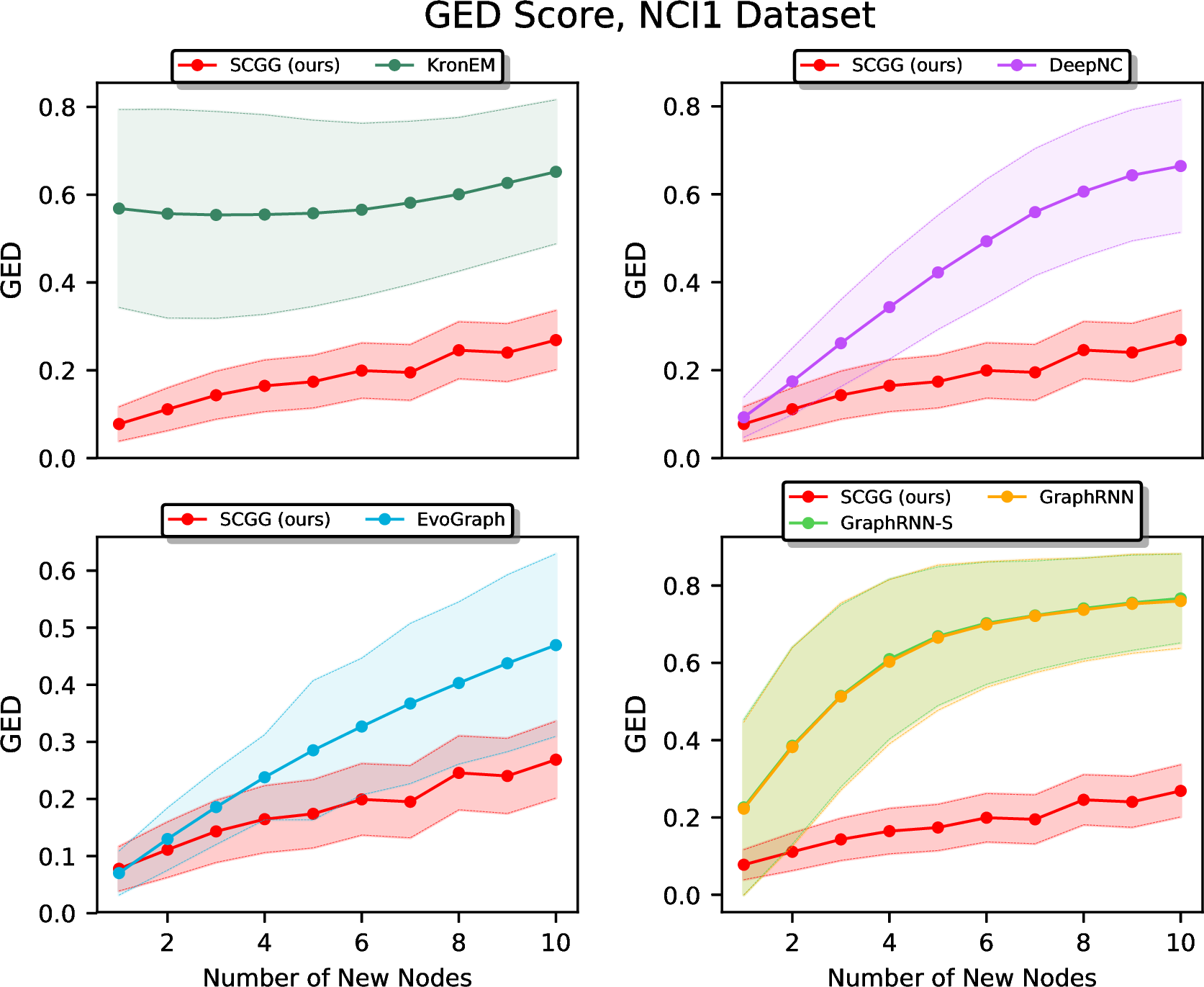

Supplement: S5 Fig — The results are reported in terms of GED (the lower the better) as a function of the number of new nodes (denoted by m) that are added to initial graphs (each represented by the notation G0 in the paper). (TIF) [file pone.0277887.s005.tif]

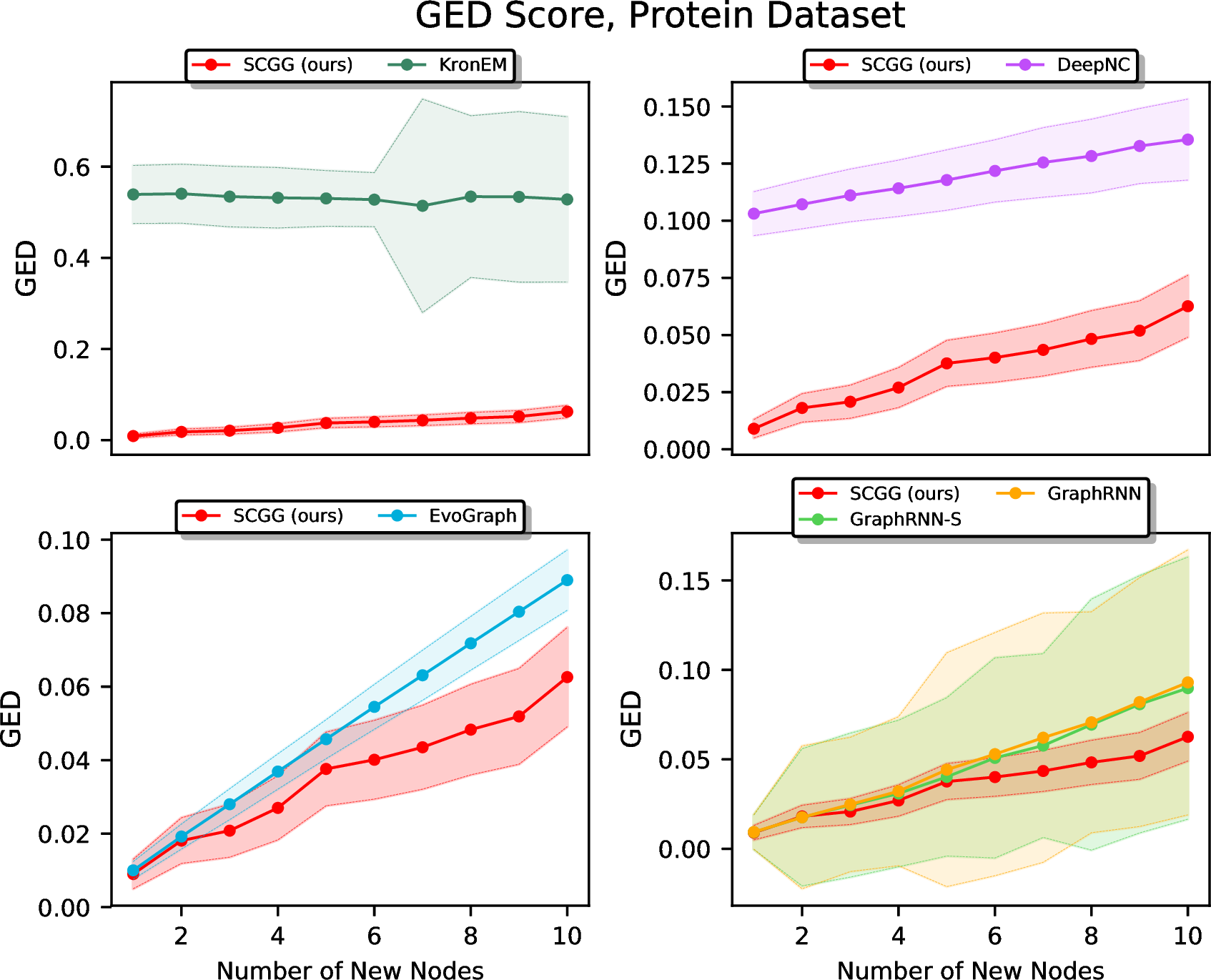

Supplement: S6 Fig — The results are reported in terms of GED (the lower the better) as a function of the number of new nodes (denoted by m) that are added to initial graphs (each represented by the notation G0 in the paper). (TIF) [file pone.0277887.s006.tif]

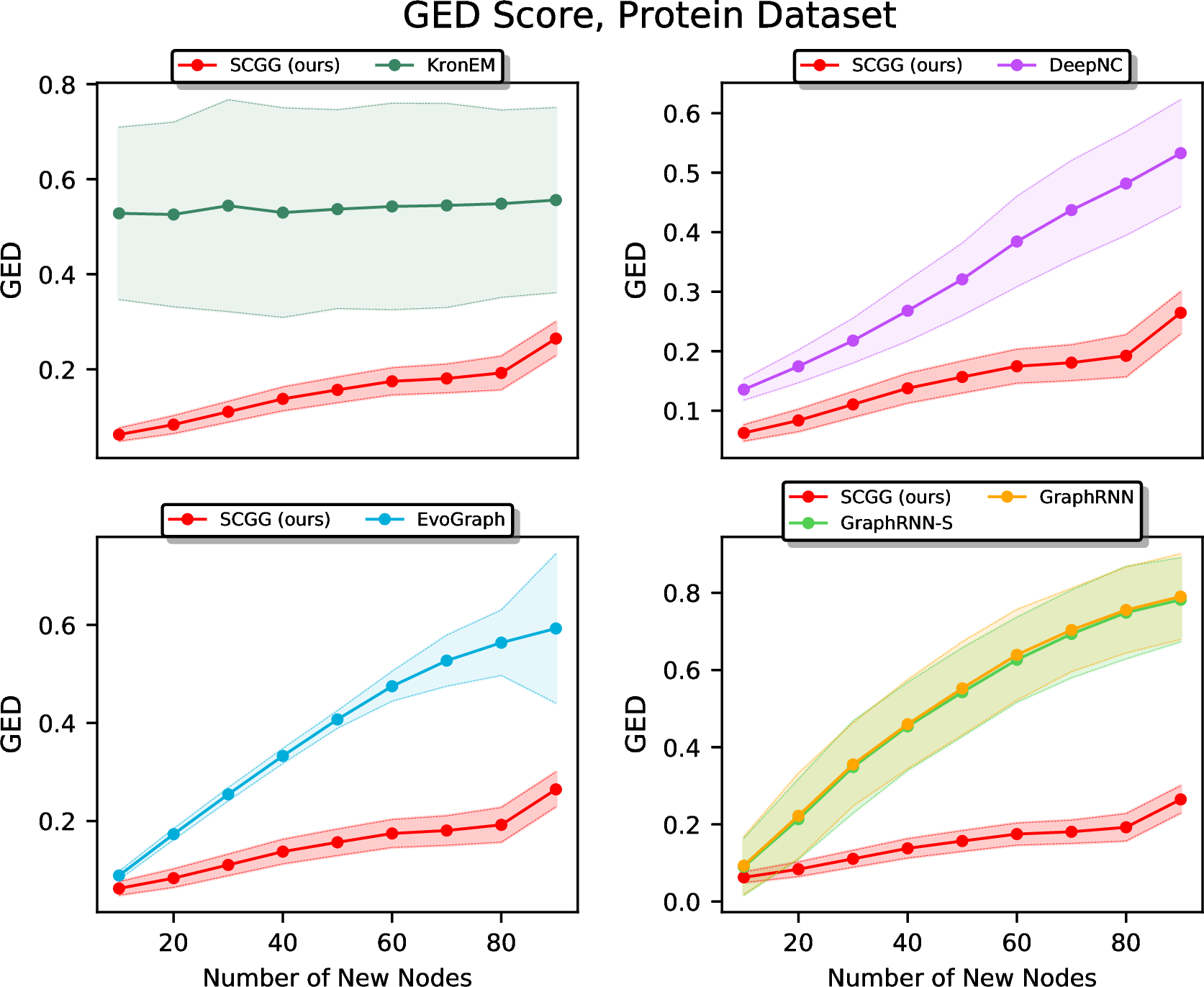

Supplement: S7 Fig — The results are reported in terms of GED (the lower the better) as a function of the parameter m that increases discretely from 10 to 90 in steps of 10. (TIF) [file pone.0277887.s007.tif]
