# Supplementary material for: SCGG: A deep structure-conditioned graph generative model
Source: PLoS One. 2022 Nov 21;17(11):e0277887. doi: 10.1371/journal.pone.0277887 (PMC9678307; doi:10.1371/journal.pone.0277887)
Supplement: S1 File — (PDF) [file pone.0277887.s008.pdf]

Hyperparameter Tuning

The hyperparameters of our SCGG model are set after development tests on validation data. In this regard, for the embedding size of the first GCN layer, we choose between 16, 32, and 64. This is also the case for choosing the embedding size of the second GCN layer. Besides, the number of the encoder layers of the Transformer is selected from 1, 2, 4, and 8. Moreover, the number of its attention heads is selected among 4, 8, and 16. For the Transformer dropout, we examine the values of 0.1, 0.2, 0.3, 0.4, and 0.5. Furthermore, since the architecture of our model, especially its recurrent neural network, shares similarities with the one proposed in [1], the decision about the remaining settings is made the same as what is recommended by the authors of [1]. To be more precise, we use 4 layers of GRU cells with a 128-dimensional hidden state to implement the function f_{RNN}. For the function f_{out}, a two-layer multilayer perceptron (MLP) is employed with 64 hidden units in the middle and a ReLU nonlinearity between the layers. Further, the model is trained with a minibatch size of 32.

References

[1] Jiaxuan You, Rex Ying, Xiang Ren, William Hamilton, and Jure Leskovec. Graphrnn: Generating realistic graphs with deep auto-regressive models. In *International conference on machine learning*, pages 5708–5717. PMLR, 2018.
