# Supplementary material for: SCGG: A deep structure-conditioned graph generative model
Source: PLoS One. 2022 Nov 21;17(11):e0277887. doi: 10.1371/journal.pone.0277887 (PMC9678307; doi:10.1371/journal.pone.0277887)
Supplement: S2 File — (PDF) [file pone.0277887.s009.pdf]

Remarks on the Order of the Number of Graph Edges

Let us denote the graph sparsity by $0 \leq x \leq 1$, which indicates the proportion of a graph's edges that do not exist. Therefore, we can denote the ratio of the available edges to all possible ones by $y = 1 - x$. Here, we want to obtain a lower bound for y to ensure that $|E|$ (i.e., the number of graph edges) is at least of order $\mathcal{O}(n)$, where n represents the number of graph nodes. As we know, the total number of possible edges of a graph is $\binom{n}{2} = \frac{n \times (n-1)}{2}$. Therefore, the following inequality should hold:

$$y \times \frac{n \times (n-1)}{2} \geq \lambda \times n \tag{1}$$

where λ is just a coefficient. We can simplify the previous inequality as follows:

$$y \geq \frac{2\lambda}{n-1} \tag{2}$$

which is the case in many graphs, including the ones in our datasets. For example, the average graph sparsity of the Protein dataset is 0.98, and therefore $y = 1 - 0.98 = 0.02$. Considering that the average number of graph nodes in this dataset is 257, and assuming that $\lambda = 1$, y must be greater than or equal to $\frac{2}{256} = 0.008$, as it is. Similar statistics are provided in the table below for all the used datasets in our paper.

Dataset	y	Lower Threshold of y
Grid	0.03	0.014
IMDBBINARY	0.49	0.111
IMDBMULTI	0.56	0.118
Enzymes	0.13	0.061
NCI1	0.08	0.069
Protein	0.02	0.008
