# Supplementary material for: SCGG: A deep structure-conditioned graph generative model
Source: PLoS One. 2022 Nov 21;17(11):e0277887. doi: 10.1371/journal.pone.0277887 (PMC9678307; doi:10.1371/journal.pone.0277887)
Supplement: S3 File — (PDF) [file pone.0277887.s010.pdf]

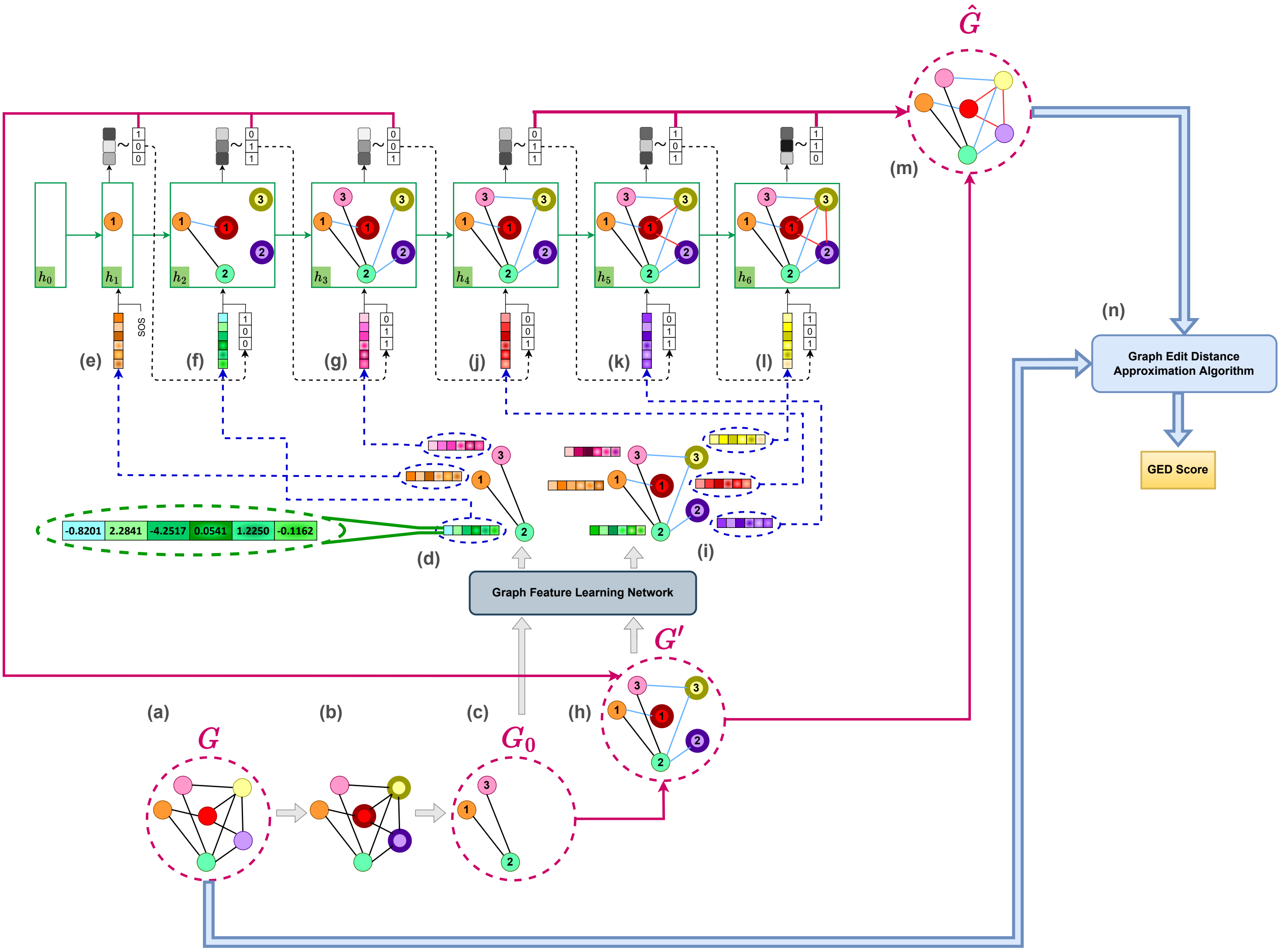

Figure 1: An illustrative example of the SCGG model at the evaluation time. (a) A graph G in the test set. (b) Randomly selecting $m = 3$ nodes from the graph G to be further treated as the set of new nodes. (c) Removing the set of new nodes from the graph G to obtain the initial graph G_0, and then applying an ordering π_n on the G_0's nodes. (d) The continuous representations of the G_0's nodes computed by the Graph Feature Learning Network. In this toy example, the dimension of node features is set to 6, where the left half of the features correspond to those computed by the GCN, and the right half (i.e., the squares colored with radial gradients) are computed by the Transformer network. For clarity, we include a magnification of one of the nodes' features (i.e., the features of the green node). (e) Generating the inter-links between the first node of G_0 (i.e., the orange node) and the set of new nodes. (f) Generating the inter-links between the second node of G_0 (i.e., the green node) and the set of new nodes. (g) Generating the inter-links between the third node of G_0 (i.e., the pink node) and the set of new nodes. (h) Building the graph G' on top of the graph G_0 by taking into account the intra-links generated in the past three steps. (i) The representations of the G''s nodes computed by the Graph Feature Learning Network. The features of the new nodes will be fed into the RNN in the following steps. (j) Generating the intra-links between the first new node (i.e., the one in red color) and the set of new nodes. (k) Generating the intra-links between the second new node (i.e., denoted by the purple color) and the set of new nodes. (l) Generating the intra-links between the third new node (i.e., the yellow node) and the set of new nodes. (m) The graph generated by the SCGG model. (n) Computing the GED score between the test graph G and the one generated by the SCGG approach using a Graph Edit Distance approximation algorithm.
