# Supplementary material for: SCGG: A deep structure-conditioned graph generative model
Source: PLoS One. 2022 Nov 21;17(11):e0277887. doi: 10.1371/journal.pone.0277887 (PMC9678307; doi:10.1371/journal.pone.0277887)
Supplement: S4 File — (PDF) [file pone.0277887.s011.pdf]

Some Potential Extensions of the SCGG Model

As discussed in the paper, our SCGG model, like many other graph generation approaches, decides based on the structural information of graphs (i.e., how graph nodes are connected) and does not consider different types of graphs or additional information associated with them. However, there exist various varieties of graphs. For example, heterogeneous graphs are an important category of graphs whose nodes and edges are of multiple types. Molecular graphs are another noteworthy example with significant applications in the pharmaceutical industry. Here, we briefly outline some potential extensions of the SCGG model so that it can be applied to other types of graphs.

At first, we give some insights on expanding the SCGG model to handle heterogeneous graphs. To start, it is necessary to consider the heterogeneity of the initial graph, which is given as a structural condition to the model. We, therefore, need to use graph neural networks specifically designed for this type of graphs to learn continuous representations of the nodes in the conditioning substructure. [1, 2, 3] provide successful examples of such methods. In the next step, it is necessary to determine the types of nodes and edges when generating new nodes and their associated edges. Although graph generation methods have rarely investigated heterogeneity, the ideas presented in [4] can be inspiring for this part of the work.

As a second example, we discuss how to expand the SCGG model so that it becomes capable of generating molecular graphs. First, we should mention that this type of conditional graph generation provides a valuable means for designing molecules. This is because the presence of specific substructures in the final chemical structure of a molecule confers a series of chemical properties to that molecule. Next, we should note that in a molecular graph, the nodes represent the atoms of the molecule and the edges represent the chemical bonds between atoms. Therefore, labels of nodes and edges determine types of atoms and chemical bonds, respectively. Due to this, as discussed earlier, our model must be able to consider types of nodes and edges when performing the graph representation learning phase. Accordingly, as a straightforward solution, we can use existing techniques such as [5] for representation learning of molecular graphs. Furthermore, it is essential to predict types of new nodes and edges in the generation process, which can be easily addressed in a way similar to the idea proposed in [6]. A crucial point that should be kept in mind when generating molecular graphs is that generator methods must ensure that the resulting molecules are valid. In addition, they should guarantee that

the molecules satisfy the desired chemical properties. We can take inspiration from [6], in which the authors employ reinforcement learning to incline their model towards generating molecules of desired characteristics. The authors of [6] also adopt a valency-based rejection sampling technique during inference to guarantee samples' validity.

References

[1] Chuxu Zhang, Dongjin Song, Chao Huang, Ananthram Swami, and Nitesh V Chawla. Heterogeneous graph neural network. In *Proceedings of the 25th ACM SIGKDD international conference on knowledge discovery & data mining*, pages 793–803, 2019.

[2] Huiting Hong, Hantao Guo, Yucheng Lin, Xiaoqing Yang, Zang Li, and Jieping Ye. An attention-based graph neural network for heterogeneous structural learning. In *Proceedings of the AAAI conference on artificial intelligence*, volume 34, pages 4132–4139, 2020.

[3] Jianxin Li, Hao Peng, Yuwei Cao, Yingtong Dou, Hekai Zhang, Philip Yu, and Lifang He. Higher-order attribute-enhancing heterogeneous graph neural networks. *IEEE Transactions on Knowledge and Data Engineering*, 2021.

[4] Chen Ling, Carl Yang, and Liang Zhao. Deep generation of heterogeneous networks. In *2021 IEEE International Conference on Data Mining (ICDM)*, pages 379–388. IEEE, 2021.

[5] Zhaoning Yu and Hongyang Gao. Molecular representation learning via heterogeneous motif graph neural networks. In *International Conference on Machine Learning*, pages 25581–25594. PMLR, 2022.

[6] Mariya Popova, Mykhailo Shvets, Junier Oliva, and Olexandr Isayev. Molecularrnn: Generating realistic molecular graphs with optimized properties. *arXiv preprint arXiv:1905.13372*, 2019.
